# Nutritional Properties and Preventive Potential of *Leptadenia hastata* (Pers) Decne (Apocynaceae) Against Metabolic Diseases

**DOI:** 10.1002/fsn3.70468

**Published:** 2025-07-04

**Authors:** Adjaratou Souratié, Crépin I. Dibala, Hemayoro Sama, Roger Dakuyo, Kayaba Kaboré, Abdoudramane Sanou, Mamounata Diao, Mamoudou Hama Dicko

**Affiliations:** ^1^ Laboratory of Biochemistry, Biotechnology, Food Technology and Nutrition (LABIOTAN) University of Joseph Ki‐ZERBO Ouagadougou Burkina Faso; ^2^ Virtual University of Burkina Faso Ouagadougou Burkina Faso; ^3^ University Daniel Ouezzin Coulibaly Dédougou Burkina Faso

**Keywords:** antioxidant, *Leptadenia hastata*, nutritional potential, oxidative stress

## Abstract

Oxidative stress is implicated in various age‐related pathologies, including cardiovascular, ocular, and neurodegenerative diseases. Dietary intake of natural antioxidants can bolster the body's defenses against oxidative damage. This study investigated the nutritional and antioxidant potential of *Leptadenia hastata* leaves, a food and medicinal plant from Burkina Faso. Standard analytical methods were used to study the physicochemical, nutritional and, antioxidant potential of the leaves. Analysis revealed that *L. hastata* leaves are rich in nutrients. per 100 g, 71.18 ± 1.83 g of dry matter, 6.89 ± 0.065 g of total ash, 1.67 ± 0.02 g of protein, 11.85 ± 0.33 g of sugar and 1.90 ± 0.07 g of lipid, 60.81 ± 2.31 mg of vitamin C. The water content was 82.59 ± 0.45 g per 100 g of dry leaves. The leaves also showed interesting levels of essential amino acids, including lysine (9.17%), leucine (8.51%), valine (5.61%), phenylalanine (5.29%) and isoleucine (4.44%). In addition, *L. hastata* leaves showed interesting lycopene (2.70 ± 0.08 mg lycopene/100 mg DM) and β‐carotene (2.99 ± 0.09 mg β‐carotene/100 mg DM) contents. Total phenol content varied from 13.95 ± 0.61 to 36.21 ± 1.88 mg EAG/100 mg while total flavonoid content varied from 0.42 ± 0.11 to 1.95 ± 0.09 mg EQ/100 mg fraction. The ethyl acetate fraction showed the highest concentration of these compounds. Furthermore, the leaves demonstrated strong antioxidant activity in DPPH and FRAP assays, correlating with their phenolic content. These findings suggest that *Leptadenia hastata* is a valuable source of nutrients, bioactive compounds, and natural antioxidants. Its potential as a dietary supplement for combating oxidative stress‐related diseases warrants further exploration.

## Graphical abstract

*Leptadenia hastata* leaves are rich in essential amino acids, vitamin C, β‐carotene, and lycopene. The ethyl acetate fraction showed the highest antioxidant activity, with strong DPPH and FRAP values correlated with phenolic content. These results support its potential as a dietary source of natural antioxidants for preventing oxidative stress‐related metabolic diseases.

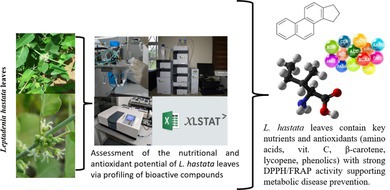

## 1 Introduction

Poor eating habits and a relatively sedentary lifestyle in developing countries contribute to the production of reactive oxygen species (ROS) and free radicals in the human body. Of course, the formation of reactive oxygen species (ROS) is important for cell regulation and homeostasis (cell signaling, apoptosis, etc.). However, when the body's endogenous antioxidant defenses (the enzymatic and non‐enzymatic defense system) are dysfunctional, these species can become toxic by reacting with cellular constituents (nucleic acids, proteins, lipids); this state is known as oxidative stress (Farhat 2019). Oxidative stress is potentially one of the causes of the onset of various pathologies such as cardiac and neurodegenerative diseases, joint pathologies, and carcinogenesis (Kowalczyk et al. 2021). Many recent clinical and experimental studies show that oxidative stress plays a central role in the progression of type 2 diabetes, obesity and metabolic liver disease, contributing to insulin resistance, chronic inflammation and endothelial dysfunction (Dawi et al. 2025; H. Li et al. 2023; Weinberg Sibony et al. 2024).

To protect against these diseases, plant‐derived dietary antioxidants play an essential preventive role here. Edible fruits, vegetables and medicinal plants contain antioxidant polyphenols, vitamins and minerals capable of scavenging ROS, inhibiting lipid peroxidation and regenerating antioxidant enzyme systems (Vicente et al. 2022). Non‐timber forest products (NTFPs), and in particular traditional leafy vegetables, are the subject of renewed interest for their potential contribution to the nutritional prevention of non‐communicable diseases in sub‐Saharan Africa (Meinhold and Darr 2019; Min et al. 2024). Among the NTFPs studied, *Leptadenia hastata* (Pers.) Decne (Apocynaceae) is a climbing plant widely distributed in semi‐arid areas of Africa. It is traditionally used as a leafy vegetable and has a long history of medicinal use (Hama et al. 2019). Its nutritional importance has been highlighted by several ethnobotanical studies, which rank it among the preferred species in rural diets (Thiombiano et al. 2010; Ayessou et al. 2018). In medicine, *L. hastata* leaves are used in the traditional treatment of hypertension, asthma, coughs, inflammatory pain and diabetes, due to their richness in active principles (Abdulsalam et al. 2020; Muhammad et al. 2023; Zhang et al. 2024). In addition, it is used for the treatment of hypertension, catarrh, pain and inflammation (Dambatta and Aliyu 2011), and also in the treatment of diabetes (Bello et al. 2011), etc. Some sources attribute good antioxidant properties to it (Sanda et al. 2023). However, despite its widespread use and many reported medicinal properties, few studies have systematically documented the nutritional characteristics and antioxidant potential of *L. hastata* leaves in an integrated approach linking bioactivity and prevention of metabolic diseases. A better understanding of its biochemical and nutritional properties could contribute to its valorization as a functional food and health resource in vulnerable communities. It is in this context that the present study aims to characterize the nutritional composition and antioxidant potential of *Leptadenia hastata* (Pers.) Decne leaves, with a view to assessing its interest in the prevention of metabolic diseases linked to oxidative stress.

## 2 Material and Methods

### 2.1 Plant Material

The plant material consisted of fresh leaves of *Leptadenia hastata* (Pers) Decne (Apocynaceae) collected in Burkina Faso, specifically in Ouagadougou, in the Bangreweogo urban park (12°23′51.492″N‐1°29′35.016″W). For identification and authentication, we used the services of Dr Lassina TRAORE, botanist at the Laboratory of Biology and Ecology of the University of Ouagadougou. A reference specimen bearing the number 1148/03/2023/CID was de‐posited in the herbarium of the Life and Earth Sciences Unit, University of Ouagadougou.

### 2.2 Methods

#### 2.2.1 Sample Collection

After collection, the leaves were dried under ventilation at room temperature (20°C–25°C). After drying, the leaves were ground into flour in a microanalysis mill to pass a 0.5 mm sieve. Grinding was carried out at 4°C, and care was taken to avoid overheating. The leaf powders were stored at −20°C before analysis to avoid endogenous enzymatic or non‐enzymatic reactions after the powders had been subjected to various extraction solvents.

#### 2.2.2 Characterization of the Nutritional Potential of Leaves

##### 2.2.2.1 Extraction and Determination of Protein Content

A 100 mg mass of the sample powder was homogenized in 1 mL of 0.1% NaCl. The mixture was then centrifuged at 4,000 rpm for 15 min and the supernatant collected was used to estimate protein content (Kielkopf et al. 2020). The estimation of water‐soluble protein content was performed according to the assay method of (Bradford 1976). A volume of 50 μL of sample is added to 250 μL of Bradford reagent (Coomassie Brillant Blue G250). The reading is made using a spectrometer at 595 nm against a blank consisting of 50 μL of sample and 250 μL of buffer solution. BSA served as the standard for the preparation of the calibration curve. Results were expressed in mg per 100 mg of sample.

##### 2.2.2.2 Determination of Carbohydrate Content

500 mg of sample was homogenized in 5 mL of 80% hot ethanol. After cooling, the homogenate was centrifuged at 4000 rpm for 10 min. The supernatant was used to estimate the soluble sugar content of fresh leaves. The soluble sugar content was determined using the phenol‐sulfuric acid method as described by Dubois et al. (1956) and the absorbance was read at 490 nm. The total sugar content was expressed as μg glucose equivalent/100 g of sample (μg GE/100 g of sample).

##### 2.2.2.3 Determination of Total Fat Content

The total fat content of the samples was determined by gravimetric method according to the standard of AOAC (2006) using a Soxhlet apparatus (R040605, Gerhardt, Germany). Fat content was calculated using equation:

$$\mathrm{Fat}\;\text{content}\;\left(\%\mathrm{DM}\right)=\frac{W1-W0}{\mathrm{Ws}}$$

where, *W*0: Weight of the empty balloon (g); *W*1: Weight of the balloon after extraction and drying (g); and *Ws*: Initial sample weight (g).

##### 2.2.2.4 Determination of Potential Energy Value

Potential energy value was estimated using the Atwater coefficients. The calorific value of the sample is calculated (Daugherty and Watkins 1976) as follow:

Energy value=P×4Kcal+G×4Kcal+L×9Kcal=Xkcal/100g.

where *P*, *G*, *L* are the proportions of proteins, carbohydrates, and lipids, respectively.

##### 2.2.2.5 Determination of Energy Values or Energy Density

The determination of energy values (EV) is carried out using the principle of calculation with the conversion coefficients for metabolized energy, also known as Atwater's general factors.

##### 2.2.2.6 Determination of Amino Acid Content

The determination of amino acids was carried out by the PICO‐TAG method. For the determination of the complete amino acid profile, 500 mg sample was weighed and placed in a 20 mL volumetric flask. The volumetric flask is filled up to the mark with 0.1 M hydrochloric acid. Approximately 1 mL of the diluted sample is filtered through the 0.45 μm filters and then derivatized with phenylisothiocyanate (PITC) to produce the amino acids phenylthiocarbamyl (PTC). The derivatized sample were added 200 μL of PICO‐TAG dilution solution (0.38 μg/μL), the amino acid derivatives were then separated by HPLC (2.3 μg sample/μL) and detected by absorption spectrophotometry at values as lofc bbnbnw as 1 pmol using a wavelength at 254 nm (Griffin and Dean 2017).

#### 2.2.3 Potential of Leaves in Bioactive Compounds and Natural Antioxidants

##### 2.2.3.1 Determination of β‐Carotene and Lycopene Content

β‐Carotene and lycopene contents were assessed by adapting the methods described, respectively, by Sombié et al. (2019). Fresh apples and almonds (300 mg) were mixed with 3 mL of 95% ethanol. The mixture was kept for 10 min on ice and centrifuged for 1 min at 4500 rpm. For β‐ carotene and lycopene, the absorbances were read at different wavelengths, and the contents are calculated according to the following equations:

$$\text{lycopene}\;\left(\frac{\mathrm{mg}}{100\;\mathrm{mL}}\right)=-0,0458\;A_{663}+0,572\;A_{505}-0,0806\;A_{453}$$

$$\beta -\text{Carotene}\;\left(\frac{\mathrm{mg}}{100\;\mathrm{mL}}\right)=0,216\;A_{663}+0,304\;A_{505}-0,452\;A_{453}$$

##### 2.2.3.2 Preparation of Extracts

50 g of powdered plant material as described earlier were extracted with 80% aqueous ethanol (500 mL) in ratio of 1/10 (*w*/*v*) for 48 h under mechanical agitation (SM 25 shaker, Edmund BÜHLER, Germany) at room temperature (20°C–22°C). After filtration, ethanol was removed under reduced pressure in a rotary evaporator (BÜCHI, Rotavopor R‐200, Switzeland) at approximately 40°C and freeze‐dried with a Telstar Cryodos 50 freeze‐dryer. These were filtered and freeze‐dried. The extract residues were weighed before being packed in waterproof plastic flasks and stored at 4°C until use. The yields of different crude extracts were calculated and expressed as grams of extract residues/100 g of dried plant materials. Fractionation The aqueous extracts were subjected to sequential liquid‐liquid extraction with petroleum ether, dichloromethane, ethyl acetate, and n‐butanol. Each fraction was then collected and concentrated to dryness under reduced pressure to obtain hexan fraction, dichloromethane fraction, ethyl acetate fraction, and n‐butanol fraction. The fractions were freeze‐dried. The fraction residues were packed in waterproof plastic flasks and stored at 4°C until use (Dibala et al. 2016).

##### 2.2.3.3 Phenolic Content Determination

###### 2.2.3.3.1 Total Phenolic Content

Total phenolic compounds were quantified according to the procedure described by Singleton et al. (1999) modified by Hasperué et al. (2016). It is based on the high oxidizability of phenolic compounds. The colorimetric properties of the Folin–Ciocalteu Reagent (FCR) are modified when it is complexed with certain molecules. It reacts with the OH function of phenols. The absorbances are read at 760 nm with a spectrophotometer. Gallic acid is used as a reference compound.

###### 2.2.3.3.2 Total Flavonoids Content

The content of total flavonoids was performed by the colorimetric method described by Arvouet‐Grand et al. (1994). This method is based on the aluminum chloride (AlCl_3_) test. 0.5 mL of methanol from each fraction extract solution (0.1 mg/mL) was mixed with 1.5 mL of AlCl_3_ (2%) and incubated for 30 min at room temperature after this incubation period. The absorbance was read spectrophotometrically against a blank at 415 nm. The results were expressed as mg quercetin equivalent (QE) per gram of fresh material.

##### 2.2.3.4 Determination of Antioxidant Activity

Anti‐free radical activity was assessed using DPPH, which was one of the first free radicals used to study the structure–antioxidant activity relationship. The ability of the antioxidant to scavenge free radicals was evaluated by the percentage of DPPH discolorations in methanolic extracts at 1 mg/mL in triplicate. The 2,2′‐diphenyl‐1‐picrylhydrazyl method was based on a spectrophotometric measurement of the changes in concentration of the DPPH radical resulting from its reaction with an antioxidant. The ability to scavenge the DPPH radical was measured spectrophotometrically at 517 nm (Velázquez et al. 2003). The percentage of inhibition was estimated based on the discoloration (Scherer and Godoy 2009).

The antioxidant activity was determined by the ferric reducing antioxidant power (FRAP) method (Benzine 2006). The assay is based on the reduction of ferric ion (Fe^3+^) to ferrous ion (Fe^2+^) which is accompanied by the appearance of an intense blue coloration quantifiable at 700 nm (Kovalevskaya et al. 2020). A methanolic extract of concentration 1 mg/mL was used for the assay. The concentration of reducing compounds in the extract was expressed in of μg ascorbic acid equivalent (AAE/100 mg DM).

#### 2.2.4 Statistical Analysis

The data were analyzed using descriptive statistics with the Microsoft Excel software version 2018. A Fisher test at the 5% level was performed for mean comparison. For each parameter, the measurements were repeated in triplicates to minimize bias.

## 3 Results

### 3.1 Leaf Physico‐Chemical Parameters

Biochemical analyses (Table 1) of *Leptadenia hastata* leaves showed that 100 g of leaf powder contained 1.67 ± 0.02 g of protein, 11.85 ± 0.033 g of sugar, 1.90 ± 0.07 g of lipid with an energy intake of 71.18 ± 1.83 kcal. The vitamin C content was 60.81 ± 2.31 mg/100 g dry matter (DM). Total ash was 6.89 ± 0.065 g/100 g DM. The water content expressed in g per 100 g of fresh matter (in g/100 g DM) was 82.59% ± 0.45% and the percentage of dry matter (DM) was 17.41% ± 0.55%.

**TABLE 1:** Biochemical analyses of *Leptadenia hastata* leaves.

Biochemical parameters	Content
Total proteins (mg/100 mg)	1.67 ± 0.27
Carbohydrates (mg/100 mg)	11.85 ± 0.03
Lipids (g/100 g)	1.90 ± 0.07
Energy value (kcal/100 g)	71.18 ± 1.83
Vitamin C (mg/100 g)	60.81 ± 2.31
Total ash (g/100 g)	6.89 ± 0.07
Moisture (g/100 g MF)	82.59 ± 0.45

**FIGURE 1:**
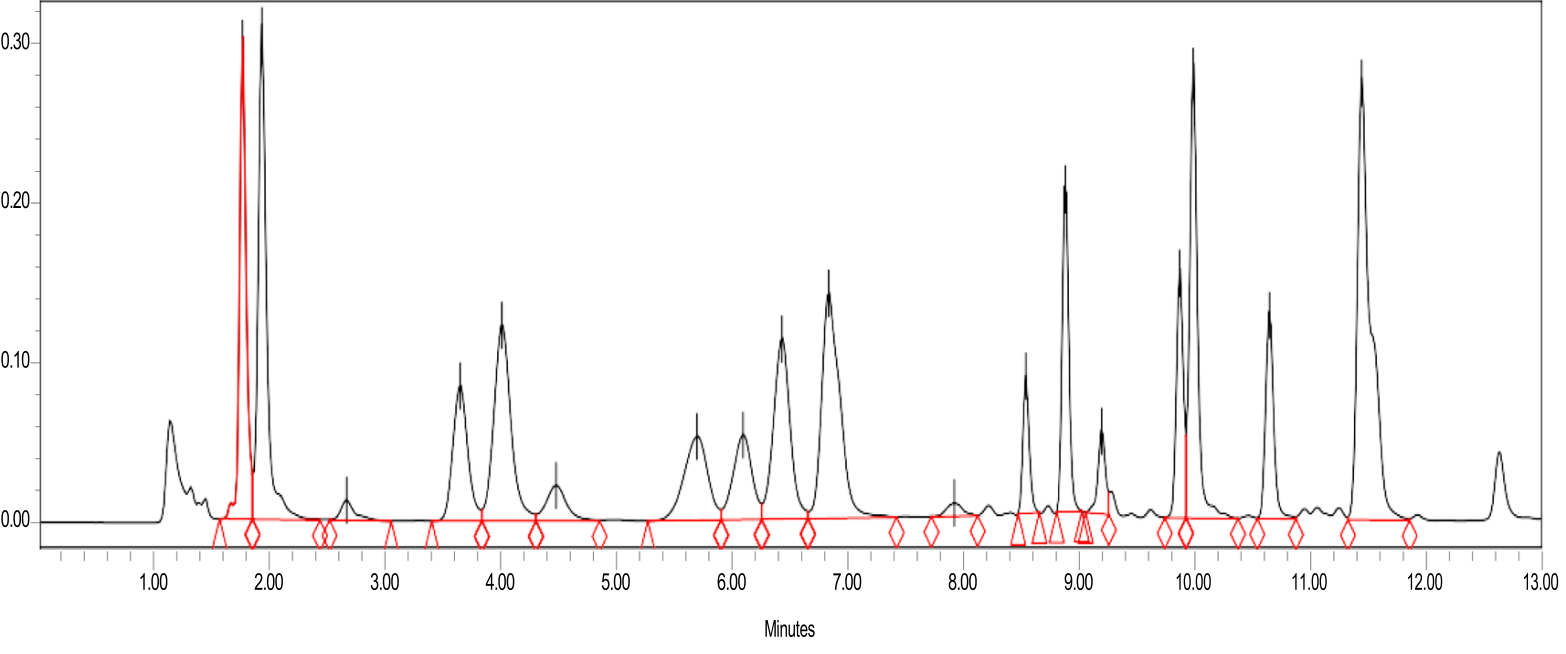
Chromatograms of amino acid profiles of *L. hastata* leaves.

**FIGURE 2:**
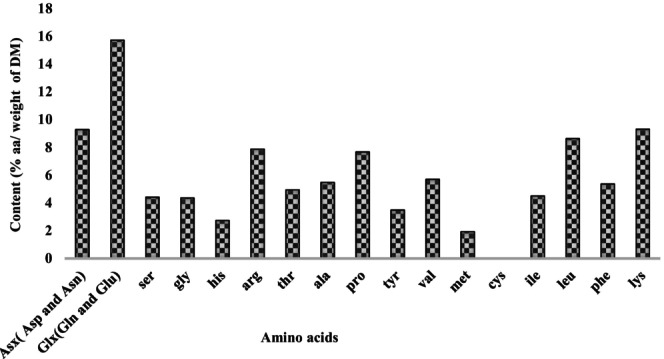
Amino acid profile of *Leptadenia hastata* leaves. The amino acid profile showed that the percentage of total non‐essential amino acids (TNEAA) was in the majority with a frequency of 54.04%, while the percentage of total sulfur amino acids (TSAA) was the lowest (1.89%). The percentage of essential amino acids was 39.81%. The aromatic essential amino acid (AEAA) content was 18.57% and the aromatic essential amino acid content was 5.29%. The total basic amino acid (TBAA) of 27.36% was higher than the total acidic amino acid (TAAA) of 7.88% (Figure 3). Histograms with different letters (a–h) are statistically different at the 5% level.

The amino acids in *Leptadenia hastata* leaves were determined by HPLC. The chromatograms obtained (Figure 1) revealed the presence of 17 peaks. Of these, 19 were quantified. The absence of glutamate (Glu) and aspartate (Asp) is explained by their transformation into glutamine (Gln) and asparagine (Asn) respectively. Levels ranged from 1.89% to 15.50% for methionine and glutamine respectively. Apart from tryptophan, all the essential amino acids lysine (9.17%), leucine (8.51%), valine (5.61%), phenylalanine (5.29%), isoleucine (4.44%), tyrosine (3.44%) and methionine (1.89%) were detected and quantified. Cystine was not detected (Figure 2).

**FIGURE 3:**
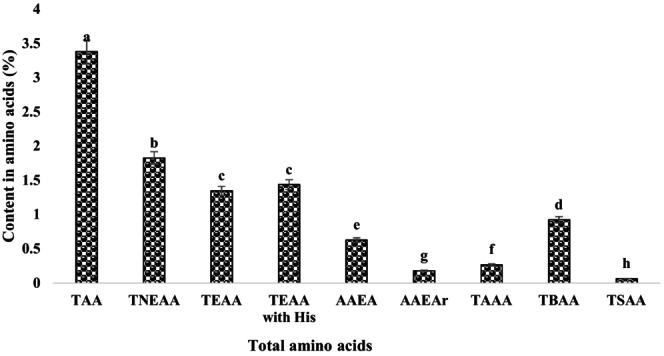
Classification of total amino acid composition of *Leptadenia hastata* leaves. AAEAr, essential Aromatic amino acids; TAA, total amino acids; TAAA, total acidic amino acids; TAAEA, essential aliphatic amino acids; TBAA, total basic amino acids; TEAA, total essential amino acids; TNEAA, total non‐essential amino acids; TSAA, total sulfur amino acids.

### 3.2 Lycopene and β‐Carotene Content

The lycopene and β‐carotene contents, expressed in μg/100 mg dry matter, were 2.70 ± 0.08 and 2.99 ± 0.09 mg dry matter for lycopene and β‐carotene respectively (Table 2).

**TABLE 2:** The lycopene and β‐carotene contents.

Parameters	Quantities (mg/100 mg)	
Carotenoids	β‐carotene	2.99 ± 0.09
	Lycopene	2.70 ± 0.08

### 3.3 Phenolics and Flavonoids Content

**FIGURE 4:**
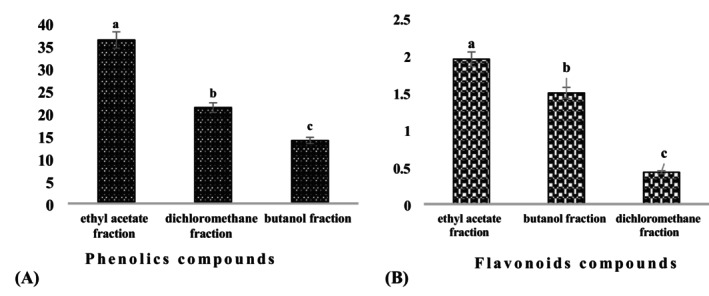
Total phenolic compound (A) and flavonoid (B) content of *Leptadenia hastata* leaves. Histograms with different letters (a–c) are statistically different at the 5% level.

The total phenol and flavonoid contents of *Leptadenia* leaf fractions, expressed as mg EAG/100 mg fraction and mg EQ/100 mg fraction respectively, are shown in Figures 4A and 4B. Our results showed a significant variation in the total phenol content of the fractions from 36.21 ± 1.88 to 13.95 ± 0.61 mg EAG/100 mg fraction. The ethyl acetate fraction of the leaves had the highest total phenol content (36.21 ± 1.88 mg EAG/100 mg fraction), followed by the dichloromethane fraction (21.24 ± 0.81 mg EAG/100 mg fraction). The lowest content was recorded in the butanol fraction (13.95 ± 0.61 mg EAG/100 mg fraction). The highest levels of total flavonoids were obtained with the ethyl acetate fraction at 1.95 ± 0.09 mg TEQ/100 mg fraction, followed by the butanol fraction at 1.49 ± 0.03 mg TEQ/100 mg fraction. The lowest content was recorded in the dichloromethane fraction (0.42 ± 0.11 mg QE/100 mg fraction).

### 3.4 Antioxidant Activity of Leaf Fractions

**FIGURE 5:**
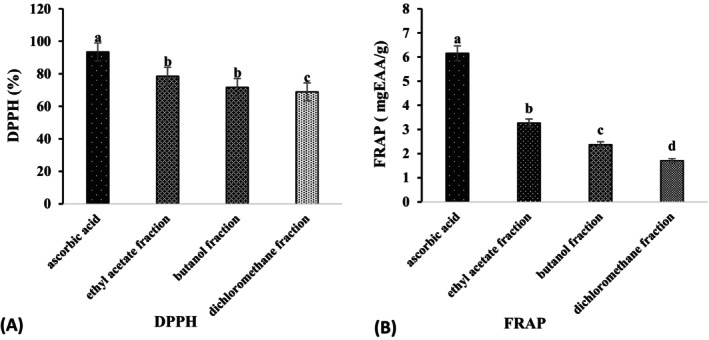
Antioxidant activity of leaf fractions: DPPH radical scavenging activity (A). Ferric reducing antioxidant power (B). Histograms with different letters (a–c) are statistically different at the 5% level.

Two methods were used to assess the oxidizing activity of the different fractions of *Leptadenia hastata* leaves. The results showed a significant variation in activity depending on the fractions. The first evaluated the DPPH antiradical activity and the second the reducing power of iron using the FRAP method. The DPPH free radical scavenging activity of our leaf fractions varied between 68.87% ± 6.81% and 78.46% ± 2.24% for a concentration of 5 mg/mL of fraction (Figure 5A). The best activity was recorded with the ethyl acetate fraction with a percentage inhibition of 78.46% ± 2.24%, followed by the butanol fraction (71.65% ± 3.13%). The lowest inhibition was recorded with the dichloromethane fraction (68.87% ± 6.81%). However, the activity of the fractions was lower than that of ascorbic acid (93.43% ± 0.19%) at the same concentration used as a standard. As for the reducing power of iron, the results of the reducing power of the fractions varied from 1.70 ± 0.02 to 3.27 ± 0.07 mg EAA/g fraction. The best reducing power was obtained with the ethyl acetate fraction (3.27 ± 0.07 mg EAA/g fraction), followed by the butanol fraction (2.37 ± 0.08 mg EAA/g faction), while the lowest content was obtained with the dichloromethane fraction (1.70 ± 0.02 mg EAA/g faction). The control (ascorbic acid) had a reducing power of 6.15 ± 0.03 mg EAA/g fraction (Figure 5B).

The Pearson correlation matrix (Table 3) between phenolic compound content, flavonoid content and antioxidant activity showed that phenolic compound content correlated positively with flavonoid content (*r* = 0.47), DPPH antiradical activity (*r* = 0.66) and FRAP iron reducing power (*r* = 0.7115) respectively. In addition, flavonoid content was positively and significantly correlated with iron reducing power (*r* = 0.95) and anti‐free radical activity determined by DPPH methods (*r* = 0.62). Iron reducing power and anti‐radical activity determined by DPPH methods were also positively correlated (*r* = 0.68).

**TABLE 3:** Pearson correlation matrix.

	Total phenol	Total flavonoids	DPPH	FRAP
Total phenol	**1**			
Total flavonoids	**0.4661**	**1**		
DPPH	**0.6553**	**0.6246**	**1**	
FRAP	**0.7115**	**0.9462**	**0.6772**	**1**

*Note:* Values in bold are different from 0 at a significance level alpha = 0.05.

## 4 Discussion

Assessment of the nutritional potential of *Leptadenia hastata* leaves has highlighted the species' interest as a nutrient‐rich plant. Analyses have shown that the leaves are a valuable source of macronutrients, notably carbohydrates and proteins, as well as essential amino acids, which are indispensable for the proper functioning of the human organism. These macronutrients play a central role in energy supply, tissue repair, and metabolic regulation. In addition, the leaves showed appreciable energy value, reinforcing their relevance as a food in both urban and rural contexts. These results confirm the traditional ethnobotanical use of *L. hastata* as an edible wild vegetable and support its integration into nutritional strategies, particularly in regions affected by protein‐energy malnutrition. The energy value was relatively higher than that reported by Hassan et al. (2020) whose work reported an energy value of 49.79 ± 1.89 kcal/100 g for the leaves. Our results show that the total ash content (6.89 ± 0.065 g/100 gMS) of the leaves is important. Ash content has a significant influence on mineral content. Our total ash content is lower than that reported by Hassan et al. (2020) (8.73% ± 0.14%) for the same type of samples. This difference in energy value could be explained by a difference in the soil and climatic conditions prevailing at the collection sites. In comparison with other tropical vegetables, the energy value of *L. hastata* leaves is close to that of Baobab leaves (74 kcal/100 g) and lower than that of Cassava (97 kcal/100 g), but higher than that of Sweet Potato (54 kcal/100 g) (Stadlmayr et al. 2012). *Leptadenia hastata* leaves showed a high content of essential amino acids, accounting for 39.81% of the total amino acid composition. Among these, lysine, leucine, phenylalanine, and valine were particularly abundant. These amino acids play a fundamental physiological role, notably in protein synthesis, immune system function, muscle metabolism, and neurotransmitter regulation, reinforcing the potential nutritional interest of this plant in human nutrition (P. Li et al. 2007; Rehman et al. 2023). Furthermore, our results indicate a predominance of basic (e.g., lysine, arginine) and aliphatic (e.g., leucine, valine) amino acids, suggesting that *L. hastata* could be a valuable source of amino acids with high nutritional value. To our knowledge, no previous studies have reported comparable data on the amino acid composition of *L. hastata* leaves, making our results of particular interest in the current literature. Further research is nevertheless required to confirm these observations and assess the bioavailability of these compounds in vivo. The extraction and fractionation yields showed that the butanol fraction presented a better extraction yield. These results highlight the richness of our extracts in polar compounds, as butanol is the most polar of the solvents used in fractionation. The *L. hastata* leaf fractions showed fairly interesting levels of total phenols and total flavonoids, particularly in the ethyl acetate fractions. The total flavonoid content was higher in the butanol fraction than in the dichloromethane fraction. This suggests that, in addition to flavonoids, the dichloromethane fraction contains other phenolic compounds (tannins and/or phenolic acids), given its higher concentration of total phenols than the butanol fraction. Our results for total phenols (13.95 ± 0.61–36.20 ± 1.88 mg EAG/100 mg fraction) and total flavonoids (0.42 ± 0.11–1.95 ± 0.09 mg EQ/100 mg fraction) obtained are relatively higher than those obtained by (Bello et al. 2011). Their total phenol and flavonoid contents varied respectively from 17.58 ± 1.55 to 37.77 ± 1.12 mg/g and 10.50 ± 0.34–15.85 ± 0.23 mg/g for the aqueous solvents, methanol and acetone. Several factors may explain these variations in our results. These include climatic conditions, the sampling period, or changes in the analytical methods used and the extraction solvents. Nevertheless, the abundance of phenolic compounds in our fractions could justify the use of the plant in traditional medicine (Umaru et al. 2018). Phenolic compounds have several biological properties, such as anti‐tumor, anti‐mutagenic, and antibacterial. These activities may be linked to their antioxidant activity. They may also act as antioxidants thanks to their ability to complex free radicals and catalyze oxidation (Umaru 2018). With the two antioxidant methods used, we found that the best activity was observed in the ethyl acetate fraction. However, this fraction showed low activity compared with ascorbic acid. In addition, our results showed that the antioxidant activity assessed by the FRAP method was strongly correlated with the content of total phenols and total flavonoids. This observation is consistent with the literature, which reports that dietary polyphenols contribute to protection against oxidative stress, a factor implicated in various chronic pathologies (Alia et al. 2024; Quesada‐Vázquez et al. 2024; Rudrapal et al. 2022). To date, few in vivo or mechanistic studies have demonstrated a direct link between *Leptadenia hastata* extracts and the modulation of glucose metabolism, lipid metabolism, or chronic inflammatory processes. However, some recent studies suggest a therapeutic potential in this field. For example, the work of Martha OrenduOche et al. (2022) evaluated the effects of a methanolic extract of *L. hastata* leaves in rats made diabetic by streptozotocin. The authors observed a moderate but significant reduction in fasting blood glucose levels, as well as a partial improvement in hepatic and renal biochemical parameters after chronic administration of the extract at different doses. These results suggest a potential hypoglycemic activity, probably mediated by antioxidant compounds. In addition to phenolics and flavonoids, the antioxidant potential of *Leptadenia hastata* leaves can also be attributed to the presence of carotenoids (lycopene and β‐carotene) and vitamin C. Our analysis revealed appreciable levels of these antioxidant micronutrients, with values of 2.70 ± 0.08 mg lycopene/100 mg and 2.99 ± 0.09 mg β‐carotene/100 mg dry matter. These concentrations are remarkable and suggest that the leaves could make a significant contribution to antioxidant defense mechanisms. It should be noted that the β‐carotene content observed in our study is significantly higher than that reported by Sena et al. (1998), who obtained a value of 50.8 μg/g dry weight, or 0.0508 mg/g. This variation could be explained by differences in plant material, environmental conditions, maturity at harvest, or the analytical methods used. These results support the hypothesis that, in addition to phenolic compounds, the presence of lipophilic antioxidants such as carotenoids enhances the plant's antioxidant profile and could justify some of its uses in traditional medicine.

## 5 Conclusion

This study highlights the nutritional and phytochemical potential of *Leptadenia hastata* (Pers.) Decne. The leaves proved to be a good source of essential nutrients such as sugars, proteins, and amino acids, with remarkable levels of lysine, leucine, phenylalanine, and valine. Although the total protein content is moderate, the amino acid profile suggests a possible contribution to the nutritional quality of diets. The high ash content and the presence of storage‐friendly compounds also indicate good post‐harvest stability. Phytochemical analysis revealed appreciable quantities of total polyphenols, flavonoids, ascorbic acid, and carotenoids, particularly lycopene and β‐carotene, with a predominance in the ethyl acetate fraction. Antioxidant assays (DPPH and FRAP) showed that the fractions possessed moderate antioxidant activity, significantly correlated with their phenolic compound content. Given its richness in nutrients and natural antioxidants, *L. hastata* could represent an interesting dietary supplement. However, these results do not point to any preventive or therapeutic action against oxidative stress‐related diseases. Future studies, including mechanistic approaches and biological models, are needed to confirm the potential effects observed.

## Author Contributions

The study protocol was drafted by Ad.S., C.I.D., H.S., and R.D. The manuscript was written by A.S., C.I.D., and M.H.D., and performed statistical analyses by Ab.S., R.D., and M.D. Scientific supervision of the study was provided by C.I.D. and M.H.D. All authors contributed to the article and approved the submitted version.

## Conflicts of Interest

The authors declare no conflicts of interest.

## Data Availability

The data used to support the findings of this study are available from the corresponding author upon request.
